# Supplementary material for: Effective and efficient content-based similarity retrieval of large lung CT images based on WSSLN model
Source: PLoS One. 2023 Sep 29;18(9):e0285573. doi: 10.1371/journal.pone.0285573 (PMC10540964; doi:10.1371/journal.pone.0285573)
Supplement: Attachment — Submitted filename: Response letter.doc [file pone.0285573.s001.doc]

Dear Editors & Reviewers,

First, thanks so much for your insightful and valuable comments which help us further improve the quality of this paper. Please review the following feedbacks.

**Q1**. *The authors need to re-write the abstract again to show the objective of this paper clearly.*

**A1**: The abstract has been updated. The objective of the paper is study of retrieving similar CT image(*CI*)s to an input one from the large-scale *CI* database of labeled diseases which is helpful to realize precise computer-aided diagnosis.

**Q2**. *Introduction section needs to be finished with summary and the contribution should be pointed out.*

**A2**: The summary and the contributions have been highlighted at the end of Section 1.

**Q3**. The proposed approach each stage is not discussed in details, nor each stage is interlink appropriately and the discussion is very poor. Please revise it carefully.

**A3**: Each stage in our proposed approach has been revised and updated carefully.

**Q4**. *The author also needs to make a clear conclusion/novelty at the end of each sub-section of methodology.*

**A4**: We have made a clear description of the contributions in each subsection of methodology.

**Q5**. *In result section, the authors require to present clear details about the data analysis.*

**A5**: The reasons and data analyses for almost all experiments have been updated in Section 4.

**Q6.** *The following recent studies on should be discussed in related work;
Content-based image retrieval and semantic automatic image annotation based on the weighted average of triangular histograms using support vector machine, 2018
Query-by-visual-search: multimodal framework for content-based image retrieval, 2020
Boosting the performance of the BoVW model using SURF–CoHOG-based sparse features with relevance feedback for CBIR*

*A novel image retrieval based on rectangular spatial histograms of visual words, 2018*

*A Novel Image Retrieval Based on a Combination of Local and Global Histograms of Visual Words, 2016*

**A6:** We have updated the related work.
